# Virtual Screening of Repurposed Drugs as Potential Spike Protein Inhibitors of Different SARS-CoV-2 Variants: Molecular Docking Study

**DOI:** 10.3390/cimb44070208

**Published:** 2022-07-04

**Authors:** Ahmad F. Eweas, Hosam-Eldin H. Osman, Ibrahim A. Naguib, Mohammed A. S. Abourehab, Ahmed S. Abdel-Moneim

**Affiliations:** 1 Department of Pharmaceutical and Medicinal Chemistry, National Research Centre, Cairo 12622, Egypt; eweas1@gmail.com; 2 Department of Science, University of Technology and Applied Sciences Rustaq, Rustaq 133, Oman; 3 Department of Anatomy, College of Medicine, Taif University, P.O. Box 11099, Taif 21944, Saudi Arabia; h.hussein@tu.edu.sa; 4 Department of Pharmaceutical Chemistry, College of Pharmacy, Taif University, P.O. Box 11099, Taif 21944, Saudi Arabia; i.abdelaal@tu.edu.sa; 5 Department of Pharmaceutics, Faculty of Pharmacy, Umm Al-Qura University, Makkah 21955, Saudi Arabia; maabourehab@uqu.edu.sa; 6 Department of Pharmaceutics, College of Pharmacy, Minia University, Minia 61519, Egypt; 7 Department of Microbiology, College of Medicine, Taif University, P.O. Box 11099, Taif 21944, Saudi Arabia

**Keywords:** SARS-CoV-2, VOC, VOI, COVID-19, variant, omicron, delta, alpha, beta

## Abstract

Like most of the RNA viruses, SARS-CoV-2 continuously mutates. Although many mutations have an insignificant impact on the virus properties, mutations in the surface protein, especially those in the receptor-binding domain, may lead to immune or vaccine escape variants, or altered binding activities to both the cell receptor and the drugs targeting such a protein. The current study intended to assess the ability of different variants of interest (VOIs) and variants of concern (VOCs) of SARS-CoV-2 for their affinities of binding to different repurposed drugs. Seven FDA approved drugs, namely, camostat, nafamostat mesylate, fenofibrate, umifenovir, nelfinavir, cefoperazone and ceftazidime, were selected based on their reported in vitro and clinical activities against SARA-CoV-2. The S1 protein subunit from eleven different variants, including the latest highly contiguous omicron variant, were used as targets for the docking study. The docking results revealed that all tested drugs possess moderate to high binding energies to the receptor-binding domain (RBD) of the S1 protein for all different variants. Cefoperazone was found to possess the highest binding energy to the RBD of the S1 protein of all the eleven variants. Ceftazidime was the second-best drug in terms of binding affinity towards the S1 RBD of the investigated variants. On the other hand, fenofibrate showed the least binding affinity towards the RBD of the S1 protein of all eleven variants. The binding affinities of anti-spike drugs varied among different variants. Most of the interacting amino acid residues of the receptor fall within the RBD (438–506).

## 1. Introduction

Severe acute respiratory syndrome coronavirus-2 (SARS-CoV-2) belongs to the family *Coronaviridae*. The structural proteins of the SARS-CoV-2 include the spike (S) protein, nucleocapsid protein (N), envelope protein (E) and matrix protein (M). The S protein is translated as an inactive precursor (S0) that requires post-translation cleavage at the furin polybasic cleavage site by the host-derived serine protease enzymes into S1 and S2 [1]. Viral neutralizing antibodies are directed to S1. S1 is also responsible for binding of the virus to the cell surface receptor. The receptor-binding domain is the part of the S1 that binds to human angiotensin converting enzyme-2, ACE2, before entering the cells by clathrin-mediated endocytosis [2]. SARS-CoV-2 was found to have ten-fold higher binding affinity to the ACE2 in comparison to other SARS-CoVs [3]. More recently, metabotropic glutamate receptor subtype 2 (mGluR2) was suggested to be an important factor for internalization of SARS-CoV-2 into the cell after cell binding to ACE2 [4].

Similar to other coronaviruses, SARS-CoV-2 mutates continuously. Most of these mutations possess an insignificant role in virus virulence or transmissibility [5]. However, a small proportion could result in the emergence of different types of variants of interest (VOI) and variants of concern (VOC). VOC are proved to be associated with increase transmissibility, virulence and/or decreased effectiveness to available diagnostics and vaccines, while VOI are variants that harbour a genetic constitution that are supposed to affect the virus transmissibility, virulence, break through immunity, diagnostics and spread in many countries. Currently, five VOC, alpha (B.1.1.7), beta (B.1.351), gamma (P.1), delta (B.1.617.2) and omicron (B.1.1.529), and two VOI, lambda (C.37) and mu (B.1.621), are circulating in different parts of the world [6]. The recent omicron VOC contains 15 amino acid substitutions in the RBD [7]. Hindering the S/ACE2 receptor binding by neutralizing antibodies or antiviral drugs could inhibit viral replication by preventing viral entry to the host cells [2,8,9]. However, the presence of accelerating genetic variation of the S1 and RBD could be a real challenge against using this type of antiviral strategy [7].

In the present study, seven repurposed FDA approved drugs were selected to investigate their potential inhibitory activities of the S1 unit RBD domain of the S1 protein of different SAR-CoV-2 variants via molecular docking. The selection of the drugs under investigation was based on their recently reported activity against SARS-CoV-2 as potential repurposed drugs. Fenofibrate, a hyperlipidaemic drug, was reported to significantly reduce SARS-CoV-2 infection in cell culture models [10]. Both camostat mesylate, used as a treatment of chronic pancreatitis, and nafamostat mesylate, an anticoagulant drug, are serine protease inhibitors that inhibit TMPRSS2. Camostat was first speculated to possess antiviral activity against SARS-CoV-2 [1]. Afterwards, camostat mesylate and nafomostat were considered as potential repurposed drugs against SARS-CoV-2 [11,12]. The anti-HIV drug nelfinavir has been reported as a potential inhibitor of the cell fusion of the SARS-CoV-2 S-glycoprotein [13]. Umifenovir is a broad-spectrum antiviral drug approved in Russia and China for treatment of influenza, SARS and Lassa viruses. In the current COVID-19 pandemic, umifenovir underwent several clinical trials as a potential repurposed drug for treatment of SARS-CoV-2 infection [14,15]. Ceftazidime is an antibiotic that inhibits SARS-CoV-2 infection in vitro. It inhibits the SARS-CoV-2 main protease and blocks the binding of SARS-CoV-2 S1 to the ACE2 [16,17]. Cefoperazone is another third-generation cephalosporin broad-spectrum antibiotic used for treatment of both mild and severe cases of SARS-CoV-2 infections in combination with sulbactam, with significant cure rates [18].

The current study intended to screen and compare the binding affinity of different potential SARS-CoV-2 antiviral agents to the S1 of omicron and other variants.

## 2. Materials and Methods

### 2.1. Ligand Preparation

**Figure 1:**
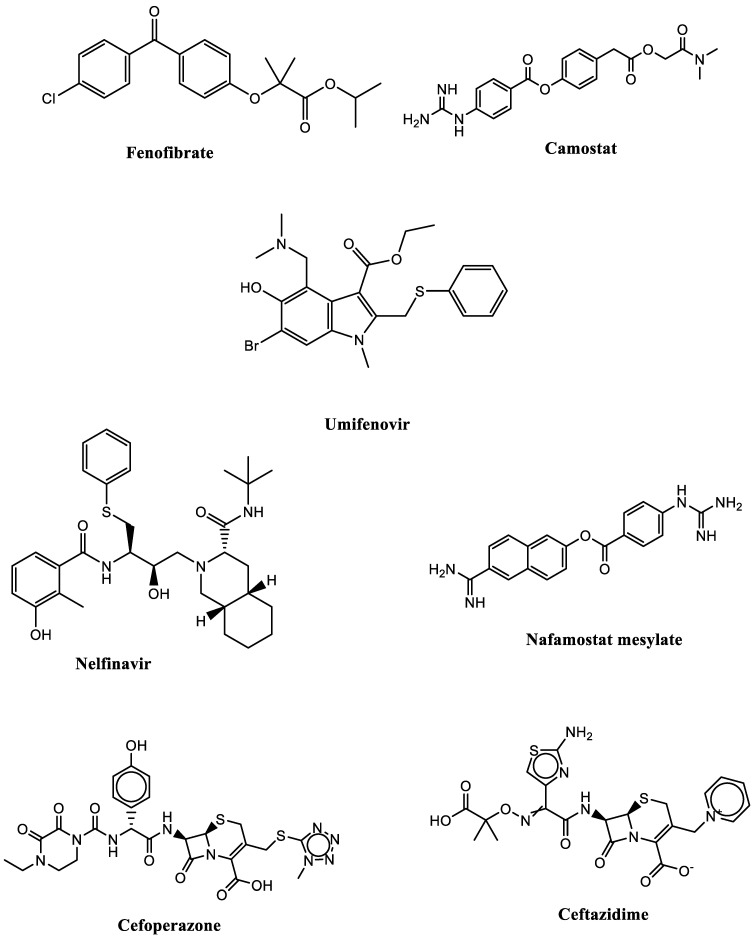
Potential SARS-CoV-2 S1 glycoprotein repurposed FDA approved drugs.

The 2D structure of all drugs used in the study, including camostat, fenofibrate, nafamostat mesylate, nelfinavir, umifenovir, cefoperazone and ceftazidime, were compiled by us using ChemDraw Professional. The 3D structures of all drug ligands were constructed using Chem 3D ultra 17.0 software molecular modelling and analysis (CambridgeSoft Corporation, Cambridge, MA, USA (2017)), then they were energetically minimized using MOPAC (semi-empirical quantum mechanics0, Job Type with 100 iterations and minimum RMS gradient of 0.01, and saved as an MDL MolFile [*.mol] (Figure 1).

### 2.2. S1 Protein Retrieval and Homology Modelling

Sequences of the S1 RBD protein of the different SARS-CoV-2 variants, including Wuhan-Hu-1 (NC-045512), alpha(GRY, B.1.1.7, EPI-ISL-679974), beta(GH, B.1.351, EPI-ISL-2447894), delta(GK, B.1.617.2, EPI_ISL_3473491), gamma(GR, P.1, EPI-ISL-3218258), lambda(GR, C37, EPI-ISL-1534645), mu(GH, UAL90205), eta(G, B.1.525, EPI-ISL-760883), iota(GH, B.1.526, EPI-ISL-3364539), kappa(G, B.1.617.1, EPI-ISL-2758215) and omicron(B.1.1.529, GR, EPI-ISL-6795850), were retrieved from GISAID and GenBank databases. Amino acid deduced amino acid sequences of the S1 protein were aligned using Mega 5 software (Supplementary Material File S1). Sequences were loaded into the SWISS-MODEL server (http//swissmodel.expasy.org/) on 9 January 2022 using the default settings of the server to create 3D homology models of the S1 protein variants. The online server created three different models of each variant. The top ranked homology models were downloaded as PDB files. The downloaded PDB files underwent protein preparation and optimization including removal of all cofactors and ligands using standard protein preparation protocol in Molecular Virtual Docker (MVD) v 6.0 software.

### 2.3. Molecular Docking

The molecular docking between the FDA approved potential S1 inhibitor drugs and SARS-CoV-2 S1 target proteins of different variants were studied using Molegro Virtual Docker (MVD, Molexus IVS, Molexus IVS, Rørth Ellevej 3, Rørth, DK-8300 Odder, Denmark) 2013.6.0 software [19]. The docking process was carried out using a 20 Å grid radius adjusted to contain amino acids residues of the S1 protein variants, which were identified as the RBD domain of SARS-CoV-2 S1 protein interacting with the ACE2 receptor [20]. The grid resolution was 0.30 Å. The number of runs for each docking process was 10, and the max iterations were 1500 with an energy threshold of 100; the maximum population size generated was 50; the maximum number of poses generated was 5. The docking score in the MVD algorithm, is presented as an arbitrary unit (MolDock Score). The best conformations for each docking process were selected based on the lowest score [21]. Gaps and insertions were detected in the omicron variant [7,22], which were described based on Wuhan-Hu-1 numbering. Amino acid numbers (Wuhan-Hu1 numbering): 339, 371, 373, 375, 417, 440, 446, 477, 478, 484, 493, 496,498, 501, and 505 of the important mutation in omicron [7], equivalent to 336, 368, 370, 372, 414, 437, 443, 474, 475, 480, 490, 493, 495, 498, and 502, respectively, in the tables and figures in the current study.

## 3. Results

Camostat showed low binding affinities to beta (−88.193) and kappa (−91.036) variants, as well as the original Wuhan strain (−94.755), with higher binding affinities to other variants ranging from −102.745 to −123.722 (Table 1, Supplementary Material File S2). Nafamostat mesylate showed the highest binding affinities to gamma (−142.398), iota (−129.082), lambda (−128.577) and mu (−127.883) VOI, but low binding affinity to beta (−83.507), alpha (−94.498), kappa (−96.805), eta (−100.437) and delta (−102.641) (Table 1, Supplementary Material File S3). Nafamostat mesylate had a higher number of hydrogen bonds, and a higher number of interacting amino acid residues with omicron, gamma and lambda variants (Table 1, Supplementary Material File S3). Camostat showed high hydrogen bonds for alpha, beta, delta, eta, kappa, and mu, as well as for the Wuhan original strain (Table 1, Supplementary Material File S2), while fenofibrate showed the lowest number of hydrogen bonds (Table 1, Supplementary Material File S4). The fenofibrate showed the lowest binding affinities to both alpha (−68.199) and beta (−75.096) variants, as well as original Wuhan strain (−88.424) variants, but considerable binding.

Nelfinavir showed good binding affinities to most of the SARS-CoV-2 variants; however, it showed the lowest binding affinity to the beta variant, followed by the alpha variant. Nelfinavir showed higher affinities to SARS-CoV-2 variants in comparison to umifenovir for the Wuhan original strain, in addition to delta, eta, iota, lambda and mu variants, while the reverse was detected with alpha and gamma variants (Table 2). Higher hydrogen bonds and more amino acids residues were detected in the interaction of nelfinavir with different variants in comparison to umifenovir (Table 2, Supplementary Material Files S5 and S6). Interestingly, umifenovir binds to the different variants with hydrogen bonds to one or two amino acids only.

**Figure 2:**
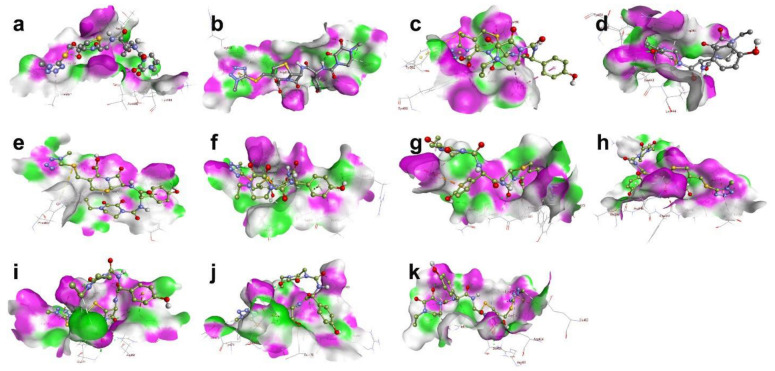
3D visualisation of the docking poses of cefoperazone to S1 subunit RBD of the different SARS-CoV-2 variants, and 3D diagrams of protein–ligand complexes, generated by BIOVIA Discovery Studio Visualiser: (**a**) Wuhan, (**b**) Alpha, (**c**) Beta, (**d**) Gamma, (**e**) Delta, (**f**) Eta, (**g**) Iota, (**h**) Kappa, (**i**) Lambda, (**j**) Mu, and (**k**) Omicron.

Cefoperazone showed the highest binding affinities to SARS-CoV-2 strains and variants, followed by the ceftazidime (Table 3). Interestingly, both cefoperazone and ceftazidime bind to Arg346, Tyr351, Asp442, Asn448 and Asn450 of the Wuhan original strain; Arg451, Lys455, Ser466 and Gln471 of the omicron variant; Tyr349, Asn448 and Arg450 of the delta variant; Ser443 Lys444, Asn450 and Tyr451 of the gamma variant; Lys458, Ser459 and Glu471 of the lambda variant; Arg458, Lys459 and Ser470 of the Mu variant; Arg346 and Leu441 of the kappa variant; Arg457, Lys458 and Ser459 of iota variant; and Glu468 and Gln471 of the eta variant (Figure 2, Supplementary Material File S7, Table 3).

The docking results revealed that all tested drugs possess moderate to high binding energies to the binding site (RBD) of the S1 protein for all different variants. Among all tested drugs, cefoperazone was found to possess the highest binding energy towards the RBD of the S1 protein of all eleven variants, ranging from a −109.913 MolDock score against the BetaGHB.1.351 variant, to a −185.011 MolDock score against the GammaGRP.1 variant. The binding interaction of cefoperazone includes multiple hydrogen bonds with the amino acid.

Residues Arg451, Arg454, Lys455, Phe456, Ser466, Ile469, Gln471 and Arg509 are common to the S1 RBD for all eleven variants. Ceftazidime was the second-best drug in terms of binding affinity towards S1 RBD of the investigated variants scoring binding energy, ranging from a −109.913 MolDock score for the BetaGHB.1.351 variant, to a −144.263 MolDock score for the GammaGRP.1 variant. On the other hand, fenofibrate showed, on average, the least binding affinity towards the RBD of the S1 protein of all eleven variants, ranging from a −68.199 MolDock score for the AlphaGRYB.1.1.7 variant, to a −114.814 MolDock score against the S1 RBD of the omicron variant. The binding interaction of fenofibrate showed, on average, three to four hydrogen bonds with the amino acid residues in the RBD of the S1 protein of the different investigated variants.

Overall, all tested repurposed drugs possess moderate to high binding affinities towards the RBD of the S1 protein subunit of all variants. Most of the interacting amino acid residues of the receptor fall within the RBD (438–506). This finding suggests that these drugs are capable of hindering the interaction of the spike protein to the human ACE2 receptor, consequently inhibiting the viral activity.

## 4. Discussion

Drug repurposing has proven to be a key strategy for finding FDA approved drugs as potential SARS-CoV-2 inhibitors, and different approaches have been used in this respect. For example, virtual screenings of FDA approved drug libraries were applied to investigate the binding energies of these drug libraries against different virus protein targets. In addition, molecular docking of specific FDA approved drugs against specific viral protein targets was also reported as a second approach to identify potential anti SARS-CoV-2 repurposed drugs. Some of the screened drugs were found to possess potential activity in-vitro, as well as in clinical studies. The spike glycoprotein was identified as a prime drug target in numerous studies using virtual screening methodologies. In this study, we focused on the potential inhibition of the selected drugs to the reported binding site of the S1 subunit of the different variants of the SARS-CoV-2 spike glycoprotein using a molecular docking approach.

In the current study, we investigated whether or not there is a variability among different virus variants in binding to different repurposed drugs. The latest VOC, omicron, possesses 15 amino acid substitutions in the RBD; these substitutions include G339D, S371L, S373P, S375F, K417N, N440K, G446S, S477N, T478K, E484A, Q493R, G496S, Q498R, N501Y and Y505H [7]. Three such amino acid substitutions, K417N, E484A and N501Y, were shared with those present in the beta (B.1.351) VOC that was dominant in South Africa [23]. Meanwhile, the gamma VOC, which spread in the Brazil and Amazon region, shared the N501Y amino acid substitution with many VOCs, including omicron and beta. It also possesses two other amino acid substitutions in the same positions in omicron, but with different amino acids: T417 and K484 [24]. Many gamma VOC strains had S477N, which increased the infectivity and spread of the virus [25]. Meanwhile, the delta variant possesses S477G (present in all VOC and VOI except iota and omicron) and T478K. Interestingly, serine at the position of 477 is frequently exposed to changes, and S477G and S477N are the most common mutations detected; both lead to increased binding to the ACE2 receptor [26]. The delta VOC possesses T478K and S477G, and was found to partially escape from neutralizing antibodies from patients vaccinated with either Pfizer or AstraZeneca vaccines, since it is neutralized 3–5-fold lower than that of the alpha VOC [27]. E484K was reported in Mu and eta, while E484Q was detected in the kappa VOC. Both mutants were found to escape neutralization by bamlanivimab [28,29]. Partial neutralization/resistance was associated with E484A/K [30,31] and Q493R [32]. Immune pressure was assumed to be responsible for the evolution of both E484K and E484Q [33]. Omicron was found to get rid of the K417N mutation [22] as it attenuates its binding to the ACE by about 4-fold [34]. N440 is an interface residue that plays a role in receptor recognition. The N440K variant is an escape mutant that was found to be evolved under selective pressure by using human monoclonal antibody C135 and showed resistance to it [35], which might increase reinfection and decrease vaccine efficiency. Such a mutant has been observed during viral passaging experiments in the presence of convalescent plasma and provides additional immune escape in vitro [4], and has been detected in 3940/17,046 omicron VOC to date. However, it is also detected in 47 isolates of delta VOC, 19 lambda VOI, 6 gamma VOC, a single beta VOC and 37 alpha VOC [36]. Interestingly, none of the 15 amino acids in the omicron variant were incorporated with the docking drugs tested in the current study; however, the neighboring amino acids in the RBD were found to be involved in the binding to the different drugs. This finding suggests that these drugs can hinder the interaction of the spike protein to the human ACE2 receptor, consequently inhibiting the viral activity. To the best of our knowledge, only two out of the seven drugs that were used in this study have been previously reported to virtually screen, via molecular docking, against the S1 of the wild-type virus. Umifenovir was found to possess considerable binding affinity to the S glycoprotein (−7.47 kcal/mol), forming a 1H bond with the Lys462 amino acid, in addition to several other hydrophobic interactions to the RBD of the S1 subunit. Meanwile, ceftazidime showed a binding affinity of −6.36 kcal/mol, forming three hydrogen bonds with LYS403, GLY504 and TYR505 amino acid residues of the RBD of the S1 protein [37,38].

Both camostat and nafamostat were found to potently inhibit SARS-CoV-2 infection in cultured human airway epithelia. Nafamostat showed a more potent effect than camostat. It also inhibited SARS-CoV-2 infection and improved disease outcomes in two COVID-19 mouse models [39]. The action is related to inhibition of the host serine protease TMPRSS2. The latter is responsible for priming of the SARS-CoV-2 S, which is necessary for virus fusion and entry to the host cell [1]. However, in our study, nafamostat was not superior to camostat, and both showed high affinities to the SARS-CoV-2 S1 protein, with slight differences of the affinities between the two compounds. The determinant of affinities is based on the SARS-CoV-2 variant type.

Fenofibrate is used as an anti-hyperlipidemic, and was found to inhibit the binding of the SARS-CoV-2 spike protein to the human ACE2 receptor and reduces the virus replication by ∼60% in Vero cells after 24 h post-infection [10]. It possesses an anti-inflammatory effect by reduction the activities of CXCL10, IL17, CCL2 and CCL20. It inhibits the phospholipid in infected cells as it possesses peroxisome proliferator-activated receptor alpha (PPARα) agonist activity, thus affecting the pathways of lipid metabolism in the lung cells of COVID-19 patients [40,41].

Dual effects on both the spike and ACE2 receptor are suggested to be among the potential antiviral activities of fenofibrate [10]. In the current study, fenofibrate showed variable activities with different SARS-CoV-2 variants, with the highest activity recorded in omicron and eta variants, and the lowest in the alpha variant. No link was found between the antiviral effect and the inhibition of cholesterol synthesis when compared with different anti-hyperlipidemic drugs. Davides et al. suggested that fenofibrate is less likely to have resistance against newly emerging strains of SARS-CoV-2; however, the current study suggested that wide variation does exist in regard to the affinity of fenofibrate to different variants [10].

Nelfinavir is a protease inhibitor that is used in the triple therapy combination used for HIV. It was found to inhibit post-entry antiviral activity against both SARS-CoV infection [42] and SARS-CoV-2 [43,44] by inhibiting the main protease. Nelfinavir mesylate might bind inside the S protein with subsequent inhibition virus entry and spike mediated cell fusion [13]. Umifenovir, known as arbidol, is involved in the reduction of the replication of SARS-CoV-2 at both viral entry and post-entry stages [45,46]. Umifenovir alone did not improve the clinical outcome of COVID-19 patients [45]. Our finding that umifenovir efficiently binds to most of the current SARS-CoV-2 variants, except the delta and beta variants, agrees with the study that reported a positive RNA test of shorter duration with umifenovir treatment in comparison to the lopinavir/ritonavir treated group [47]. Meanwhile, in comparison to the nelfinavir, the latter was found to bind more efficiently than umifenovir.

Both cefoperazone and ceftazidime are bactericidal antibiotics that contain a beta-lactam ring. The latter renders them susceptible to the beta-lactamase enzyme. Recently, ceftazidime (over 300 μM) was found to inhibit SARS-CoV-2 infection by binding to the S1 RBD of the SARS-CoV-2 [16]. In the same study, efficacy of different cephalosporins were compared, and ceftazidime was found to be more potent than other cephalosporins, including cefoperazone. However, in the current study, we found the reverse, since cefoperazone was found to more potently bind to the RBD than ceftazidime. Interestingly, high binding variabilities of cefoperazone were detected among different variants.

## 5. Conclusions

In conclusion, all tested repurposed drugs possess moderate to high binding affinities towards the RBD of the S1 protein subunit of all variants. Most of the interacting amino acid residues of the receptor fall within the RBD (438–506). The nature of the neighboring amino acids to RBD of the S1 provide important clues for the design of targeted inhibitors and/or peptidyl disruptors. Cefoperazone showed the highest binding affinities to the SARS-CoV-2 S1 protein subunit, followed by ceftazidime, nelfinavir, camostat, nafamostat mesylate and fenofibrate.

## Figures and Tables

**Table 1:** Docking of camostat, nafamostat mesylate and fenofibrate to the S1 protein of different SARS-CoV-2 variants.

Variant	Drug	Moldock Score	Protein–Ligand Interactions	H-Bonds	Interacting Amino Acids
Wuhan	Camostat	−94.755	−96.285	−11.548	Arg346, Asn448, Asn450, Tyr451
	Nafamostat mesylate	−104.846	−135.335	−9.582	Arg403, Gln409, Lys417, Try453, Asn501, Tyr505
	Fenofibrate	−88.424	−114.416	−5.119	Arg346, Asn448
Alpha	Camostat	−106.873	−120.232	−8.657	Arg451, Arg454, Ser466
	Nafamostat mesylate	−94.498	−119.399	−3.438	Arg451, Lys455
	Fenofibrate	−68.199	−100.849	−4.576	Arg451, Arg454, Ser466
Beta	Camostat	−88.193	−103.546	−10.498	Arg400, Gln490, Ser491, Gly493, Tyr498., Tyr502
	Nafamostat mesylate	−83.507	−109.470	−5.861	Gln490, Ser491, Tyr502
	Fenofibrate	−75.096	−103.255	−1.057	Arg400, Gly493, Tyr502
Gamma	Camostat	−103.073	−122.665	−7.500	Thr345, Ser443, Tyr451
	Nafamostat mesylate	−142.398	−171.688	−18.337	Thr345, Asn439, Leu441, Ser443, Asn450, Tyr451, Gln498
	Fenofibrate	−100.413	−132.544		Tyr451, Arg509
Delta	Camostat	−102.745	−125.025	−12.577	Ser347, Arg353, Arg450, Arg464
	Nafamostat mesylate	−102.641	−129.476	−10.112	Thr343, Leu439, Asn448, Tyr449
	Fenofibrate	−95.604	−130.153	−7.797	Ser347, Tyr449, Arg450
Eta	Camostat	−119.353	−137.223	−8.394	Arg451, Ser466, Gly479
	Nafamostat mesylate	−100.437	−118.492	−2.951	Lys455, Ile469, Gln471
	Fenofibrate	−112.297	−141.414	−5.000	Arg454, Lys455
Iota	Camostat	−118.221	−138.393	−5.271	Arg457, Lys458, Ser459, Ser469
	Nafamostat mesylate	−129.082	−149.932	−7.837	Arg454, Ser469, Gln471, Gln474
	Fenofibrate	−109.585	−135.721	0.000	-----
Kappa	Camostat	−91.036	−101.483	−6.946	Gly447, Asn450, Tyr451
	Nafamostat mesylate	−96.805	−120.913	−5.140	Ala352, Leu441
	Fenofibrate	−98.021	−128.495	−7.943	Ser349, Asn448, Tyr451, Arg452
Lambda	Camostat	−119.514	−144.252	-−5.005	Lys458, Thr470, Gln474, Asn481, Gly482
	Nafamostat mesylate	−128.577	−154.110	−13.652	Arg454, Ser459, Ser469, Ile472, Gln474
	Fenofibrate	−104.396	−134.550	−2.991	Ser469, Gln474
Mu	Camostat	−123.722	−148.776	−12.036	Arg455, Arg458, Lys459, Ser460, Arg467, Ser470
	Nafamostat mesylate	−127.883	−148.680	−7.204	Arg455, Ser470, Glu472, Gln475
	Fenofibrate	−103.262	−131.505	−1.108	Arg458, Lys459, Ser460
Omicron	Camostat	−115.805	−140.820	−6.926	Ser466, Thr467, Gly479
	Nafamostat mesylate	−113.863	−134.961	−11.732	Phe453, Ile469, Cys477, Gly479
	Fenofibrate	−114.814	−139.328	−3.202	Arg451, Lys455, Gln471

**Table 2:** Docking of nelfinavir and umifenovir to the S1 protein of different SARS-CoV-2 variants.

Variant	Drug	Moldock Score	Protein–Ligand Interactions	H-Bonds	Interacting Amino Acids
Wuhan	Nelfinavir	−116.080	−116.546	−11.012	Arg346, Phe347, Ser349, Asn450
	Umifenovir	−103.073	−96.644	−2.500	Tyr449
Alpha	Nelfinavir	−100.709	−121.857	−6.122	Arg454, Lys455, Ile469
	Umifenovir	−113.226	−116.466	−2.706	Arg451, Ser46
Beta	Nelfinavir	−81.228	−108.512	−12.424	Gln490, Ser491, Gln495, Tyr498
	Umifenovir	−89.495	−81.839	−2.602	Thr373, Arg405
Gamma	Nelfinavir	−130.548	−170.809	−7.209	Thr345, Arg346, Tyr451, Arg509
	Umifenovir	−135.474	−149.931	−2.500	Arg509
Delta	Nelfinavir	−104.259	−134.889	−11.305	Arg344, Phe345, Asn448, Tyr449
	Umifenovir	−92.064	−86.749	−1.436	Gln445
Eta	Nelfinavir	−128.410	−143.063	−5.233	Ile469, Gln471
	Umifenovir	−125.948	−128.545	−2.500	Asn391
Iota	Nelfinavir	−125.109	−144.609	−13.345	Arg454, Arg457, Ser459, Asp467, Ser469, Gln471
	Umifenovir	−117.812	−122.038	−2.500	Ser469
Kappa	Nelfinavir	−113.164	−133.359	−12.661	Asn354, Arg346, Phe347, Asn450, Arg452
	Umifenovir	−99.971	−102.968	−2.500	Tyr451
Lambda	Nelfinavir	−125.793	−163.020	−10.182	Lys458, Ile472, Gln474
	Umifenovir	−101.866	−116.982	−5.360	Lys458, Glu471
Mu	Nelfinavir	−137.991	−146.459	−12.536	Arg458, Lys459, Ser460, Glu466
		−124.324	−127.631	−2.500	Ser470
Omicron	Nelfinavir	−132.578	−149.554	−8.435	Arg451, Lys455, Asp464, Ser466, Glu468, Gln471
	Umifenovir	−132.726	−131.339	−4.698	Ser466, Gln471

**Table 3:** Docking of cefoperazone and ceftazidime to the S1 protein of different SARS-CoV-2 variants.

Variant	Drug	Moldock Score	Protein–Ligand Interactions	H-Bonds	Interacting Amino Acids
Wuhan	Cefoperazone	−144.371	−146.031	−18.708	Thr345, Arg346, Ser349, Tyr351, Leu441, Asp442, Asn448, Asn450, Arg509
	Ceftazidime	−118.597	−137.029	−17.625	Arg346, Tyr351, Asp442, Asn448, Asn450, Tyr451
Alpha	Cefoperazone	−125.588	−130.783	−9.288	Arg451, Arg454, Asp464, Ser466, Glu468
	Ceftazidime	−110.150	−114.360	−18.519	Arg400, Glu403, Tyr446, Tyr498, Tyr502
Beta	Cefoperazone	−109.913	−105.625	−23.514	Arg400, Ser491, Gly493, Tyr498, Tyr502
	Ceftazidime	−109.963	−128.651	−8.750	Thr373, Arg405, Tyr505
Gamma	Cefoperazone	−185.011	−179.208	−27.519	Arg346, Ser438, Ser443 Lys444, Asn450, Tyr451, Arg509
	Ceftazidime	−144.263	−179.684	−20.501	Thr345, Asp442, Ser443, Lys444, Asn450, Tyr451
Delta	Cefoperazone	−137.162	−125.012	−16.197	Tyr349, Asn448, Arg450, Thr468, Ser492
	Ceftazidime	−101.435	−146.149	−22.839	Arg344, Ser347, Tyr349, Asn448, Tyr449, Arg450
Eta	Cefoperazone	−161.781	−166.069	−14.727	Arg454, Lys455, Arg463, Glu468, Gln471
	Ceftazidime	−140.189	−155.757	−15.701	Arg451, Ser466, Glu468, Gln471
Iota	Cefoperazone	−148.607	−167.276	−11.618	Arg454, Arg457, Lys458, Ser459, Asn460, Lys462
	Ceftazidime	−135.460	−153.084	−4.952	Arg457, Lys458, Ser459, Ser469
Kappa	Cefoperazone	−146.317	−160.266	−31.556	Thr345, Arg346, Ser349, Tyr351, Leu441, Asp442, Asn450, Arg452, Arg509
	Ceftazidime	−130.048	−135.518	−15.420	Arg346, Leu441, Asn448, Tyr451
Lambda	Cefoperazone	−164.939	−167.338	−22.592	Arg454, Phe456, Arg457, Lys458, Ser459, Glu471, Gln474
	Ceftazidime	−118.877	−139.884	−12.483	Lys458, Ser459, Ser469, Glu471
Mu	Cefoperazone	−146.632	−138.655	−10.715	Arg455, Arg458, Lys459, Arg467, Ile469, Ser470, Glu472
	Ceftazidime	−123.135	−128.116	−4.888	Arg458, Lys459, Ser470
Omicron	Cefoperazone	−171.673	−178.048	−19.746	Arg451, Arg454, Lys455, Ser456, Asp464, Ser466, Gln471, Gly479
	Ceftazidime	−138.695	−155.598	−9.885	Arg451, Phe453, Lys455, Ser466, Gln471

Affinities to other variants ranged from (−95.604 to −114.814) for other variants (Table 1, Supplementary Material File S4).

## Data Availability

Not applicable.

## Supplementary Materials

Supplementary materials can be found at https://www.mdpi.com/article/10.3390/cimb44070208/s1.
