# Supplementary material for: Virtual Screening of Repurposed Drugs as Potential Spike Protein Inhibitors of Different SARS-CoV-2 Variants: Molecular Docking Study
Source: Curr Issues Mol Biol. 2022 Jul 4;44(7):3018–29. doi: 10.3390/cimb44070208 (PMC9319331; doi:10.3390/cimb44070208)
Supplement: Supplementary file 1 [file cimb-44-00208-s001.zip › Supp 7.pdf]

Supp 7

3D visualisation of the docking poses of Umifenovir to S1 subunit RBD of the different SARS-CoV-2 variants, 3D diagrams of Protein-Ligand Complexes generated by BIOVIA Discovery Studio Visualiser

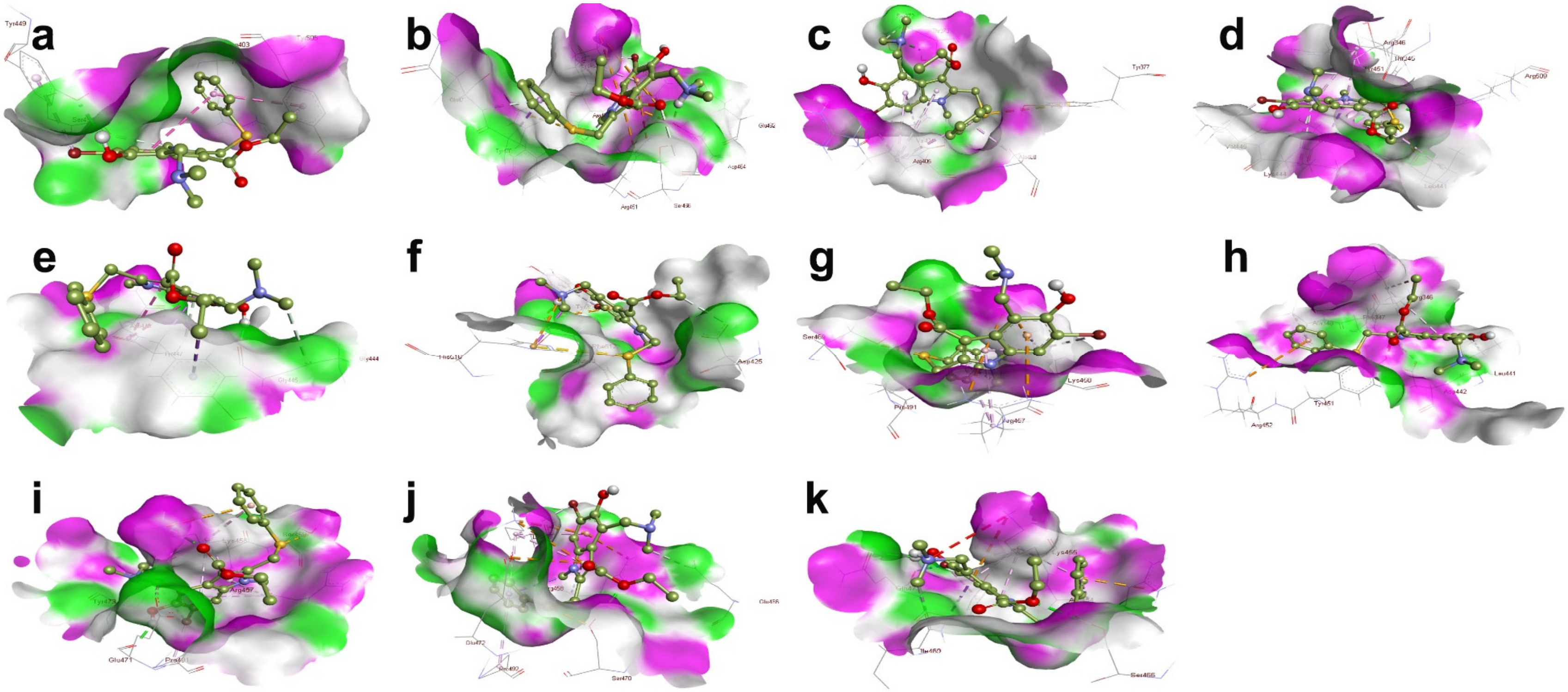

a) Wuhan, b) Alpha, c) Beta, d) Gamma, e) Delta, f) Eta, g) Iota, h) Kappa, i) Lambda, j) Mu, k) Omicron
