# Supplementary material for: Virtual Screening of Repurposed Drugs as Potential Spike Protein Inhibitors of Different SARS-CoV-2 Variants: Molecular Docking Study
Source: Curr Issues Mol Biol. 2022 Jul 4;44(7):3018–29. doi: 10.3390/cimb44070208 (PMC9319331; doi:10.3390/cimb44070208)
Supplement: Supplementary file 1 [file cimb-44-00208-s001.zip › suppl 1.pdf]

Suppl.1 Potential SARS-CoV-2 S1 glycoprotein repurposed FDA approved Drugs

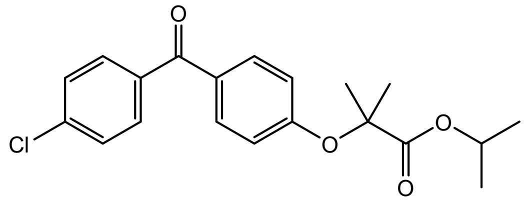

Fenofibrate

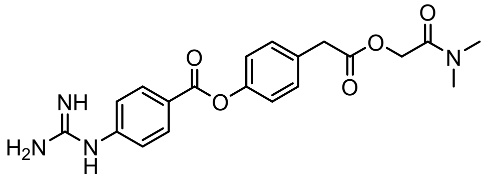

Camostat

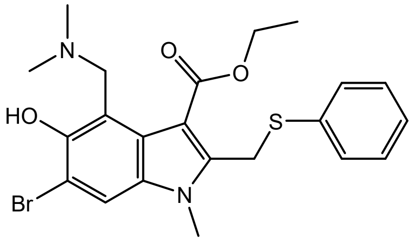

Umifenovir

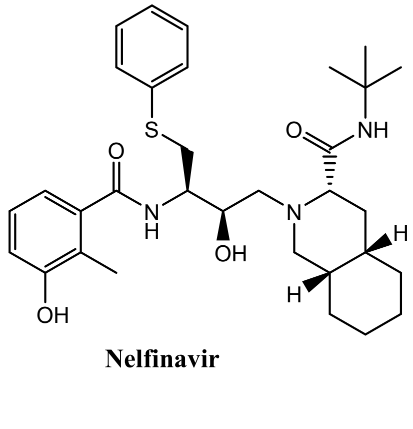

Nelfinavir

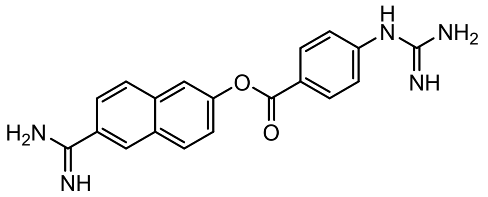

Nafamostat mesylate

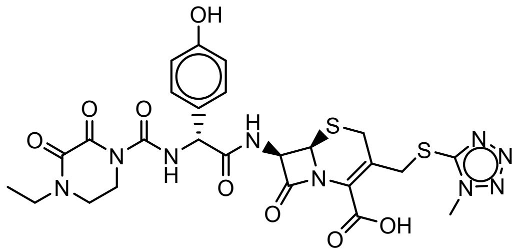

Cefoperazone

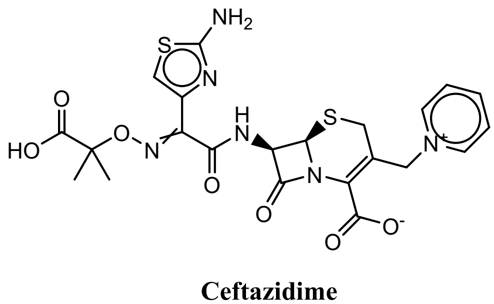

Ceftazidime
