# Supplementary material for: Virtual Screening of Repurposed Drugs as Potential Spike Protein Inhibitors of Different SARS-CoV-2 Variants: Molecular Docking Study
Source: Curr Issues Mol Biol. 2022 Jul 4;44(7):3018–29. doi: 10.3390/cimb44070208 (PMC9319331; doi:10.3390/cimb44070208)
Supplement: Supplementary file 1 [file cimb-44-00208-s001.zip › Suppl 2.pdf]

Supplement 2:

Deduced amino acid sequences of the S1 protein of the different variants used in the current study.

```
                                              |10        |20        |30        |40        |50        |60        |70        |80
NC_045512|Wuhan-Hu-1                 MFVFLVLLPL VSSQCVNLTT RTQLPPAYTN SFTRGVYYPD KVFRSSVLHS TQDLFLPFFS NVTWFHAIHV SGTNGTKRFD
Alpha|GRY|B.1.1.7|EPI_ISL_679974     .......... .......... .......... .......... .......... .......... ........-- ..........
Beta|GH||B.1.351_|EPI_ISL_2447894    .......... .......... .......... .......... .......... .......... .......... .........A
Delta|GK|B.1.617.2|EPI_ISL_3473491   .......... ........R. .......... .......... .......... .......... .......... ..........
Gamma|GR|P.1|EPI_ISL_3218258         .......... .......... .......... .......... .......... .......... .......... ..........
Lambda|GR|C37|EPI_ISL_1534645        .......... .......... .......... .......... .......... .......... .......... ..........
Mu|GH|UAL90205                       ....F..... .......... .......... .......... .......... .......... .......... ..........
Eta|G|B.1.525|EPI_ISL_760883         .......... .......... .......... .......... .......... .R........ ......V.-- ..........
Iota|GH|B.1.526|EPI_ISL_3364539      ....F..... .......F.. .......... .......... .......... .......... .......... ..........
Kappa|G|B.1.617.1|EPI_ISL_2758215    .......... .......... .......... .......... .......... .......... .......... ..........
OMICRON|B.1.1.529|GR|EPI_ISL_6795850 .......... .......... .......... .......... .......... .......... ......V.-- ..........

                                              |90        |100       |110       |120       |130       |140       |150
NC_045512|Wuhan-Hu-1                 NPVLPFNDGV YFASTEKSNI IRGWIFGTTL DSKTQSLLIV NNATNVVIKV CEFQFCNDPF LGV-YYHKNN KSWMESEFRV
Alpha|GRY|B.1.1.7|EPI_ISL_679974     .......... .......... .......... .......... .......... .......... ...--..... ..........
Beta|GH||B.1.351_|EPI_ISL_2447894    .......... .......... .......... .......... .......... .......... ...-...... ..........
Delta|GK|B.1.617.2|EPI_ISL_3473491   .......... .......... .......... .......... .......... .......... ...-...... ......G--.
Gamma|GR|P.1|EPI_ISL_3218258         .......... .......... .......... .......... .......... .......... ...-...... ..........
Lambda|GR|C37|EPI_ISL_1534645        .......... .......... .......... .......... .......... .......... ...-...... ...T......
Mu|GH|UAL90205                       .......... ....I..... .......... .......... .......... .......... ...TSN.... ..........
Eta|G|B.1.525|EPI_ISL_760883         .......... .......... .......... .......... .......... .......... ...--..... ..........
Iota|GH|B.1.526|EPI_ISL_3364539      .......... ....I..... .......... .......... .......... .......... ...-...... ..........
Kappa|G|B.1.617.1|EPI_ISL_2758215    .......... ....I..... .......... .......... .......... .......... .D.-...... ..........
OMICRON|B.1.1.529|GR|EPI_ISL_6795850 .......... ....I..... .......... .......... .......... .......... .D----.... ..........

                                     |160       |170       |180       |190       |200       |210       |220       |230
NC_045512|Wuhan-Hu-1                 YSSANNCTFE YVSQPFLMDL EGKQGNFKNL REFVFKNIDG YFKIYSKHTP INLVR---DL PQGFSALEPL VDLPIGINIT
Alpha|GRY|B.1.1.7|EPI_ISL_679974     .......... .......... .......... .......... .......... .....---.. .......... ..........
Beta|GH||B.1.351_|EPI_ISL_2447894    .......... .......... .......... .......... .......... .....---G. .......... ..........
Delta|GK|B.1.617.2|EPI_ISL_3473491   .......... .......... .......... .......... .......... .....---.. .....V.... ..........
Gamma|GR|P.1|EPI_ISL_3218258         .......... .......... .......... .......... .......... .....---.. .......... ..........
Lambda|GR|C37|EPI_ISL_1534645        .......... .......... .......... .......... .......... .....---.. .......... ..........
Mu|GH|UAL90205                       .......... .......... .......... .......... .......... .....---.. .......... ..........
Eta|G|B.1.525|EPI_ISL_760883         .......... .......... .......... .......... .......... .....---.. .......... ..........
Iota|GH|B.1.526|EPI_ISL_3364539      .......... .......... .......... .......... .......... .....---.. .......... ..........
Kappa|G|B.1.617.1|EPI_ISL_2758215    .......... .......... .......... .......... .......... .....---.. .......... ..........
OMICRON|B.1.1.529|GR|EPI_ISL_6795850 .......... .......... .......... .......... .......... .-I..EPE.. .......... ..........
```

```
                                        |240       |250       |260       |270       |280       |290       |300       |310
NC_045512|Wuhan-Hu-1                 RFQTLLALHR SYLTPGDSSS GWTAGAAAYY VGYLQPRTFL LKYNENGTIT DAVDCALDPL SETKCTLKSF TVEKGIYQTS
Alpha|GRY|B.1.1.7|EPI_ISL_679974     .......... .......... .......... .......... .......... .......... .......... ..........
Beta|GH||B.1.351_|EPI_ISL_2447894    ....---... .......... .......... .......... .......... .......... .......... ..........
Delta|GK|B.1.617.2|EPI_ISL_3473491   .......... .......... .......... .......... .......... .......... .......... ..........
Gamma|GR|P.1|EPI_ISL_3218258         .......... .......... .......... .......... .......... .......... .......... ..........
Lambda|GR|C37|EPI_ISL_1534645        .......... .......... .......... .......... .......... .......... .......... ..........
Mu|GH|UAL90205                       .......... .......... .......... .......... .......... .......... .......... ..........
Eta|G|B.1.525|EPI_ISL_760883         .......... .......... .......... .......... .......... .......... .......... ..........
Iota|GH|B.1.526|EPI_ISL_3364539      .......... ......G... .......... .......... .......... .......... .........V ..........
Kappa|G|B.1.617.1|EPI_ISL_2758215    .......... .......... .......... .......... .......... .......... .......... ..........
OMICRON|B.1.1.529|GR|EPI_ISL_6795850 .......... .......... .......... .......... .......... .......... .......... ..........

                                        |320       |330       |340       |350       |360       |370       |380       |390
NC_045512|Wuhan-Hu-1                 NFRVQPTESI VRFPNITNLC PFGEVFNATR FASVYAWNRK RISNCVADYS VLYNSASFST FKCYGVSPTK LNDLCFTNVY
Alpha|GRY|B.1.1.7|EPI_ISL_679974     .......... .......... .......... .......... .......... .......... .......... ..........
Beta|GH||B.1.351_|EPI_ISL_2447894    .......... .......... .......... .......... .......... .......... .......... ..........
Delta|GK|B.1.617.2|EPI_ISL_3473491   .......... .......... .......... .......... .......... .......... .......... ..........
Gamma|GR|P.1|EPI_ISL_3218258         .......... .......... .......... .......... .......... .......... .......... ..........
Lambda|GR|C37|EPI_ISL_1534645        .......... .......... .......... .......... .......... .......... .......... ..........
Mu|GH|UAL90205                       .......... .......... .........K .......... .......... .......... .......... ..........
Eta|G|B.1.525|EPI_ISL_760883         .......... .......... .......... .......... .......... .......... .......... ..........
Iota|GH|B.1.526|EPI_ISL_3364539      .......... .......... .......... .......... .......... .......... .......... ..........
Kappa|G|B.1.617.1|EPI_ISL_2758215    .......... .......... .......... .......... .......... .......... .......... ..........
OMICRON|B.1.1.529|GR|EPI_ISL_6795850 .......... .......... ..D....... .......... .......... ....L.P.F. .......... ..........

                                        |400       |410       |420       |430       |440       |450       |460       |470
NC_045512|Wuhan-Hu-1                 ADSFVIRGDE VRQIAPGQTG KIADYNYKLP DDFTGCVIAW NSNNLDSKVG GNYNYLYRLF RKSNLKPFER DISTEIYQAG
Alpha|GRY|B.1.1.7|EPI_ISL_679974     .......... .......... .......... .......... .......... .......... .......... ..........
Beta|GH||B.1.351_|EPI_ISL_2447894    .......... .......... N......... .......... .......... .......... .......... ..........
Delta|GK|B.1.617.2|EPI_ISL_3473491   .......... .......... .......... .......... .......... .....R.... .......... ..........
Gamma|GR|P.1|EPI_ISL_3218258         .......... .......... .......... .......... .......... .......... .......... ..........
Lambda|GR|C37|EPI_ISL_1534645        .......... .......... .......... .......... .......... .......... .......... ..........
Mu|GH|UAL90205                       .......... .......... .......... .......... .......... .......... .......... ..........
Eta|G|B.1.525|EPI_ISL_760883         .......... .......... .......... .......... .......... .......... .......... ..........
Iota|GH|B.1.526|EPI_ISL_3364539      .......... .......... .......... .......... .......... .......... .......... ..........
Kappa|G|B.1.617.1|EPI_ISL_2758215    .......... .......... .......... .......... .......... .....R.... .......... ..........
OMICRON|B.1.1.529|GR|EPI_ISL_6795850 .......... .......... N......... .......... ...K.....S .......... .......... ..........
```

```
                                        |480       |490       |500       |510       |520       |530       |540       |550
NC_045512|Wuhan-Hu-1                 STPCNGVEGF NCYFPLQSYG FQPTNGVGYQ PYRVVVLSFE LLHAPATVCG PKKSTNLVKN KCVNFNFNGL TGTGVLTESN
Alpha|GRY|B.1.1.7|EPI_ISL_679974     .......... .......... ....Y..... .......... .......... .......... .......... ..........
Beta|GH||B.1.351_|EPI_ISL_2447894    .......K.. .......... ....Y..... .......... .......... .......... .......... ..........
Delta|GK|B.1.617.2|EPI_ISL_3473491   .K........ .......... .......... .......... .......... .......... .......... ..........
Gamma|GR|P.1|EPI_ISL_3218258         .......... .......... .......... .......... .......... .......... .......... ..........
Lambda|GR|C37|EPI_ISL_1534645        .......... .......... .......... .......... .......... .......... .......... ..........
Mu|GH|UAL90205                       .......K.. .......... ....Y..... .......... .......... .......... .......... ..........
Eta|G|B.1.525|EPI_ISL_760883         .......K.. .......... .......... .......... .......... .......... .......... ..........
Iota|GH|B.1.526|EPI_ISL_3364539      N......... .......... .......... .......... .......... .......... .......... ..........
Kappa|G|B.1.617.1|EPI_ISL_2758215    .......Q.. .......... .......... .......... .......... .......... .......... ..........
OMICRON|B.1.1.529|GR|EPI_ISL_6795850 NK.....A.. ......R..S .R..Y...H. .......... .......... .......... .......... K.........

                                        |560       |570       |580       |590       |600       |610       |620       |630
NC_045512|Wuhan-Hu-1                 KKFLPFQQFG RDIADTTDAV RDPQTLEILD ITPCSFGGVS VITPGTNTSN QVAVLYQDVN CTEVPVAIHA DQLTPTWRVY
Alpha|GRY|B.1.1.7|EPI_ISL_679974     .......... ...D...... .......... .......... .......... .......G.. .......... ..........
Beta|GH||B.1.351_|EPI_ISL_2447894    .......... .......... .......... .......... .......... .......G.. .......... ..........
Delta|GK|B.1.617.2|EPI_ISL_3473491   .......... .......... .......... .......... .......... .......G.. .......... ..........
Gamma|GR|P.1|EPI_ISL_3218258         .......... .......... .......... .......... .......... .......G.. .......... ..........
Lambda|GR|C37|EPI_ISL_1534645        .......... .......... .......... .......... .......... .......G.. .......... ..........
Mu|GH|UAL90205                       .......... .......... .......... .......... .......... .......G.. .......... ..........
Eta|G|B.1.525|EPI_ISL_760883         .......... .......... .......... .......... .......... .......G.. .......... ..........
Iota|GH|B.1.526|EPI_ISL_3364539      .......... .......... .......... .......... .......... .......G.. .......... ..........
Kappa|G|B.1.617.1|EPI_ISL_2758215    .......... .......... .......... .......... .......... .......G.. .......... ..........
OMICRON|B.1.1.529|GR|EPI_ISL_6795850 .......... .......... .......... .......... .......... .......G.. .......... ..........

                                        |640       |650       |660       |670       |680  |S2
NC_045512|Wuhan-Hu-1                 STGSNVFQTR AGCLIGAEHV NNSYECDIPI GAGICASYQT QTNSPRRARS
Alpha|GRY|B.1.1.7|EPI_ISL_679974     .......... .......... .......... .......... ....H.....
Beta|GH||B.1.351_|EPI_ISL_2447894    .......... .......... .......... .......... ..........
Delta|GK|B.1.617.2|EPI_ISL_3473491   .......... .......... .......... .......... ....R.....
Gamma|GR|P.1|EPI_ISL_3218258         .......... .......... .......... .......... ..........
Lambda|GR|C37|EPI_ISL_1534645        .......... .......... .......... .......... ..........
Mu|GH|UAL90205                       .......... .......... .......... .......... ....H.....
Eta|G|B.1.525|EPI_ISL_760883         .......... .......... .......... .......... H.........
Iota|GH|B.1.526|EPI_ISL_3364539      .......... .......... .......... .......... ..........
Kappa|G|B.1.617.1|EPI_ISL_2758215    .......... .......... .......... .......... ....R.....
OMICRON|B.1.1.529|GR|EPI_ISL_6795850 .......... ........Y. .......... .......... ..K.H.....
```
